# Editorial: Advances in biotechnological applications of extreme microorganisms

**DOI:** 10.3389/fmicb.2023.1276435

**Published:** 2023-09-05

**Authors:** Kattia Núñez-Montero, Leticia Barrientos, Zeinab G. Khalil, Angelina Lo Giudice

**Affiliations:** ^1^Facultad de Ciencias de la Salud, Instituto de Ciencias Aplicadas, Universidad Autónoma de Chile, Temuco, Chile; ^2^Centre of Chemistry and Drug Discovery, Institute for Molecular Bioscience, University of Queensland, Brisbane, QLD, Australia; ^3^Department of Earth System Sciences and Technologies for the Environment, Institute of Polar Sciences, National Research Council (CNR), Venice, Italy

**Keywords:** extreme environment, extremozymes, extremophile, bacteria, omics analyses, biotechnology

In the vast tapestry of life on Earth, microorganisms inhabit every niche, often defying the limits of what seems possible. Their resilience and adaptability have intrigued scientists for generations, and recent research has unveiled their immense potential in the realm of biotechnology opening new horizons for innovation. This Research Topics delves into exciting recent discoveries made in the field of biotechnological applications of extreme microorganisms. Hence, the collection of seven articles presented in this Research Topic showcases an array of recent advancements in the biotechnological applications of extreme microorganisms. Encompassing different themes such as core microbiomes inhabiting the rhizosphere of plants from extreme environments, rhizobia's nitrogen-fixing abilities, salt-tolerant microorganism metabolism, bioactive compound discovery, industrial enzyme potential and groundwater microbial communities in remediation, these studies collectively contribute to the unfolding narrative of harnessing extreme microbes for biotechnological applications.

Study and usage of extreme microorganisms for enhancing crop resilience in the face of climate change and sustainable agriculture is proposed in two articles of this Research Topic. The study presented by Shen et al. uncovers the hidden potential of rhizobia in barren environments. This research shows the nitrogen-fixing capacity of rhizobia from *Pongamia pinnata* inhabiting the barren landscapes of vanadium-titanium magnetite tailings. Their results not only highlight the diversity of rhizobia in such extreme conditions but also hints at their potential for bioremediation and sustainable agricultural practices. On the other hand, Contreras et al. unravel the hidden connections between arid ecosystems and plant resilience. They hypothesized that microbial taxa mediating desiccation and drought stress tolerance may be shared across distant landscapes. The results described the core microbiome in diverse plant species from the Antarctic and the Atacama Desert, which could provide candidates for plant resilience to drought. Other authors explored the application against phytopathogenic fungi. By combining metabolomics, genomics, and bioassays, Maimone et al., study the biosynthetic potential of the Antarctic strain *Pseudomonas* sp. So3.2b, describing a wealth of bioactive metabolites and bioactivity against *Rhizoctonia solani*, suggesting promising applications in modern agriculture as a safer alternative to synthetic pesticides.

The potential of extreme microbes extends to the realm of industrial enzymes. A novel thermostable alkaline lipase, Lip54q, derived from a compost metagenomic library is characterized by Li et al.. With an affinity for alkaline conditions, this enzyme demonstrates stability and efficacy in various detergent formulations, with potential to enhance the detergent industry through its improved capabilities in removing oil stains. Meanwhile, the work of Rahimnahal et al. describes a novel keratinolytic protease from *Bacillus licheniformis* (KRLr1). This study dissects the enzyme's properties, catalytic efficiency, and substrate interactions, underscoring its promise across various biotechnological applications, from waste management to protein engineering for the production of valuable compounds.

Additionally, understanding the ecological and molecular traits of the extreme microbial life could contribute as groundwork for potential breakthroughs in the development of functional applications. In this context, Cai et al. studied the salt-tolerant marine probiotic *Meyerozyma guilliermondii* GXDK6, unraveling its ability to perceive and respond to salt stress. Through an integrative omics approach, they uncover an intricate network of metabolic regulations that underlie GXDK6's resilience, which include the modulation of drug metabolites. Finally, Zhu et al. characterized the groundwater microbial communities in a decommissioned acid *in-situ* Uranium mine, discovering that pH and redox potential are the dominant environmental factors affecting the microbial composition. This study provides new insights into microbial dynamics within this extreme environment and its potential for bioremediation strategies and environmental restoration.

The articles presented in this editorial collectively showcase the remarkable progress made in understanding and harnessing the potential of extreme microorganisms for diverse biotechnological applications. From agricultural resilience to industrial innovation and environmental restoration, these studies outline a future where microorganisms from previously untapped unique environments become agents of positive change. As we continue to unravel the mysteries of the microbial extreme life, we might uncover new solutions to some of the most imperative challenges confronting humanity.

## Author contributions

KN-M: Conceptualization, Writing—original draft. LB: Writing—review and editing. ZK: Writing—review and editing. AL: Writing—review and editing.

